# Research and design of internal meshing gear pump separating crescent plate

**DOI:** 10.1038/s41598-024-53892-6

**Published:** 2024-02-12

**Authors:** Shanxin Guo, Guangchi Yu

**Affiliations:** 1 College of Electronics and Information Science, Fujian Jiangxia University, Fuzhou, 350108 China; 2 Fuzhou University Hydraulic Component Factory, Fuzhou University, Fuzhou, 350002 China

**Keywords:** Energy science and technology, Engineering

## Abstract

The design of the crescent block is a key factor in the high-pressure operation of the internal meshing gear pump. In order to increase the output pressure of the pump, this article designs a new type of separable crescent plate. Then, taking a certain type of high-pressure internal meshing gear pump as the research object, a nonlinear differential equation for the internal flow field of the gear pump was established, and the pressure distribution law in the transition zone of a cycle was derived. A mathematical model of the device was established based on the equilibrium conditions of the internal and external crescent block forces. Finally, experimental research was conducted on the design parameters of the separation crescent plate. The results showed that under the conditions of displacement of 100.5 ml/r, pressure of 20.5 MPa, and rotational speed of 1800 RPM, the compensation chamber angle range was 31.23°, and the pump's volumetric efficiency could reach 94.6%. There were no abnormal phenomena during the entire operation of the pump, and there was no jamming or jamming of the friction pair.

## Introduction

Internal gear pumps have the characteristics of compact structure, small volume, low noise, and low flow pulsation, and are widely used in industrial equipment^1^. Due to its good suction performance, high energy density, and high conversion efficiency, it is more suitable for hydraulic circuits such as power systems, steering systems, and transmission systems. Due to the existence of radial imbalance force^2^, internal gear pumps are constrained in terms of pressure increase, and there is still a gap in pressure compared to plunger pumps. Therefore, the development and design of high-pressure internal gear pumps are still an important research direction in the current gear pump field^3^.

In order to further improve the pressure of internal meshing gear pumps, mainstream scholars have proposed two ideas^4^. One is dedicated to the study of how to directly reduce radial imbalance force, typical work can be seen in references^5–7^, and the other is to design a static pressure compensation groove structure to offset radial imbalance force, detailed reference can be made in references^8–10^. Because the radial imbalance forces of these two approaches cannot be completely eliminated, they are often combined with static pressure compensation structures. The compensation device designed according to this method has a crescent-shaped external structure, called a crescent block. Due to the influence of multiple complex dynamic pressures in the transition zone^11^, it is difficult to establish an accurate mathematical model. References^9,12,13^ conducted simulation research on the pressure distribution flow field on its outer surface, while references^11,14,15^ conducted experimental research on the lubrication performance of the static pressure device. However, these studies do not provide strong guidance for engineering design.

Based on these reasons, this article proposes a separated crescent block structure suitable for high-pressure internal meshing gear pumps. Then, taking this structure as the research object, numerical calculation methods and theoretical research methods suitable for solving unbalanced radial force under high-pressure working conditions of internal meshing gear pumps were explored to study the pressure distribution law in the transition zone. Combined with the compensation effect of static pressure support on unbalanced radial force, the influence of the position and structural size of static pressure support groove on the compensation effect of unbalanced radial force was analyzed. Finally, Further determine the separated crescent block structure that meets the requirements for unbalanced radial force. Meanwhile, this article conducts a series of experimental studies on the design and operation of high-pressure internal meshing gear pumps to verify the reliability of numerical calculation methods. Therefore, the actual goal of this work is to deeply study the calculation model of the radial compensation mechanism of the internal meshing gear pump.

## Working principle and key technologies

**Figure 1:**
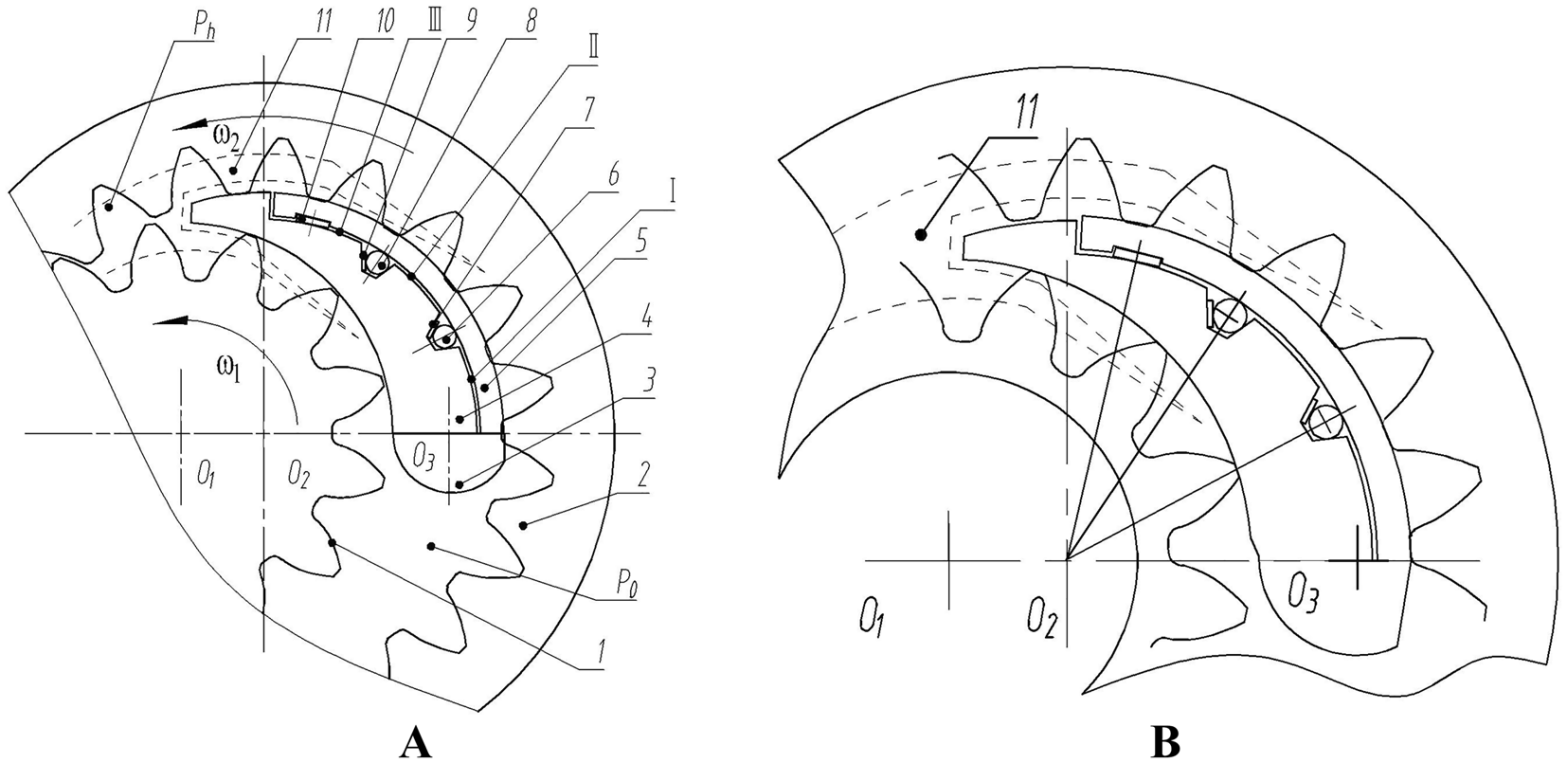
Schematic diagram of radial compensation mechanism for internal meshing gear pump: (**A**) Schematic diagram; (**B**) Partial enlarged view. (1, External gear; 2, Internal gear; 3, Stop pin; 4, The inner layer of a crescent-shaped isolation plate; 5,The outer layer of the crescent-shaped isolation plate; 6, First sealing rolle; 7, First pressure plate spring; 8, Second sealing roller; 9, Second pressure plate spring; 10, Third pressure plate spring; 11, Expanding the oil chamber).

The compensation mechanism for radial clearance of internal meshing gear pump is shown in Fig. 1.The outer gear 1 and inner gear 2 rotate counterclockwise. An oil suction cavity ${{\text{P}}}_{0}$ is formed on the lower side of the central axis, and an oil discharge cavity ${{\text{P}}}_{{\varvec{h}}}$ is formed on the upper side of the central axis. The high and low pressure areas are separated by isolation devices^16^. The isolation device consists of a stationary stop pin 10, a floating outer crescent block 9, and an inner crescent block 3. The outer surface of outer crescent block 9 fits with the tooth top circle of inner gear 2, while the inner surface of inner crescent block 2 fits with the tooth top circle of outer gear 1^17^. The first baffle spring 5, first sealing rod 6, second baffle spring 7, and second sealing rod 8 are arranged between the outer crescent block and the inner crescent block, forming zones I, II, and III^18^. Introduce the high-pressure ${{\text{P}}}_{{\varvec{h}}}$ of the oil discharge chamber into Zone I through the rectangular pressure spring 4.The transition pressure from ${{\text{P}}}_{{\varvec{h}}}$ to ${{\text{P}}}_{0}$ is distributed on the outer surface of the isolation device. By reasonably arranging the positions of the first baffle spring 5, first sealing rod 6, second baffle spring 7, second sealing rod 8, and rectangular compression spring 4, the crescent block has compensation in the diameter direction, which can compensate for surface wear between the isolation device and the gear pair^19^. It can be thus obvious that the key to increasing the pressure of the internal meshing gear pump is to achieve the balance of the compensation device under high pressure. It is necessary to prevent the increase of leakage caused by insufficient balance and the decrease of volumetric efficiency, as well as to prevent excessive balance from damaging the oil film layer and causing wear and burn^20^.

### Pressure distribution in the transition zone

The pressure in the transition zone consists of three parts: the high-pressure zone, the tooth tip clearance zone and the tooth concave zone. After adopting the technology of expanding the high-pressure area, the tooth concave area only has one tooth concave range. The pressure ${p}_{2}$ variation of the tooth concave depends on the flow difference between the radial clearance ${h}_{i}$ and the axial clearance ${h}_{f}$ per unit time^21^. During the working process, the radial clearance ${h}_{i}$ and axial clearance ${h}_{f}$ are both very small, and the working oil has a certain viscosity.^22^ Therefore, clearance flow can be considered as laminar motion. The leakage amount of the clearance can be calculated based on theoretical pressure difference shear flow model for clearance flow between two parallel plates^23,24^.

### Differential equation for pressure in tooth concave area

**Figure 2:**
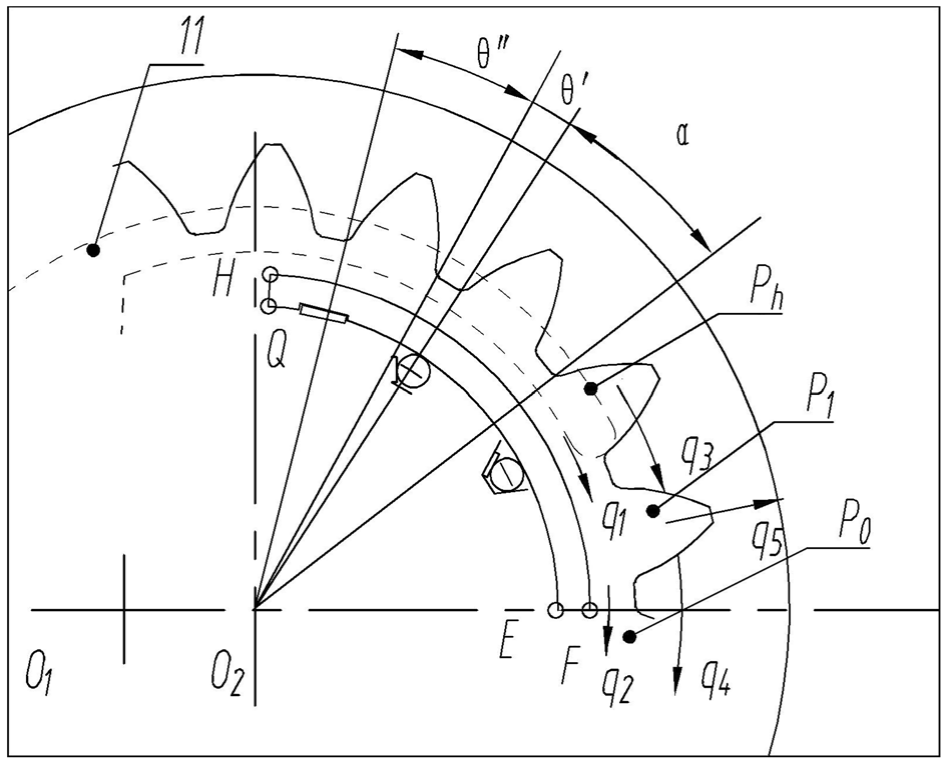
Geometric model and fluid dynamics model of the tooth concave area.

Establish a geometric model of the tooth concave area, as shown in Fig. 2. The radial clearance leakage flow consists of two parts: the leakage flow ${q}_{1}$ from the high-pressure area to the tooth concave area and the leakage flow ${q}_{2}$ from the tooth concave area to the low-pressure area.The axial clearance leakage flow consists of three parts: the leakage flow ${q}_{3}$ flowing from the high-pressure area on both sides of the gear teeth to the concave area, the leakage flow ${q}_{4}$ flowing from the concave area to the low-pressure area, and the leakage flow ${q}_{5}$ flowing from the concave area to the bearing cavity along both sides of the tooth root.

Based on the structural analysis between the gear and the crescent block and the side plate^25^, a pressure difference shear flow model was used to calculate the radial leakage flow ${q}_{1}$ and ${q}_{2}$ of the gear pump, as well as the axial clearance leakage flow ${q}_{3}$, ${q}_{4}$ and ${q}_{5}$, as follows^26^:

1 $${\text{q}}_{1} = \frac{{bh_{2i}^{3} }}{{12\mu {\text{s}}_{e2} }}\left( {p_{h} - p_{2} } \right) - \frac{{bh_{2i} u_{02} }}{2}$$

2 $${\text{q}}_{2} = \frac{{bh_{2i}^{3} }}{{12\mu {\text{s}}_{e2} }}\left( {p_{2} - p_{0} } \right) - \frac{{bh_{2i} u_{02} }}{2}$$

3 $${\text{q}}_{3} = \frac{{R_{e2} - R_{i2} }}{{6\mu {\text{s}}_{2} }}h_{2f}^{3} \left( {p_{h} - p_{2} } \right) - \left( {R_{e} - R_{i} } \right)h_{2f} u_{12}$$

4 $${\text{q}}_{4} = \frac{{R_{e2} - R_{i2} }}{{6\mu {\text{s}}_{2} }}h_{2f}^{3} \left( {p_{2} - p_{0} } \right) - \left( {R_{e} - R_{i} } \right)h_{2f} u_{12}$$

5 $${\text{q}}_{5} = \frac{{\theta_{2} h_{2f}^{3} }}{{6\mu ln\frac{{R_{i} }}{{R_{z2} }}}}\left( {p_{2} - p_{0} } \right)$$

In the equation, $\mu$ is the dynamic viscosity of hydraulic oil, $b$ is the width of the gear, ${\text{s}}_{e2}$ is the tooth tip thickness of the internal gear, ${\text{s}}_{2}$ is the width of the internal gear indexing circle, $p_{g}$ is the end pressure of the high-pressure oil tank, $p_{2}$ is the pressure in the transition zone, $p_{0}$ is the low-pressure chamber pressure, $R_{e2}$ is the radius of the top circle of the internal gear teeth, $R_{i2}$ is the radius of the inner gear root circle, $u_{02}$ is the linear velocity of the inner gear tooth tip, $u_{12}$ is the linear speed of the internal gear indexing circle, $R_{z2}$ is the radius of the internal gear shaft, $\theta_{2}$ is the included angle of the inner gear root.

Set the flow into and out of the transition zone as $q_{in}$ and $q_{out}$ , the starting point of the time when the entire tooth concave completely enters the transition zone is ${ }t = 0$ , and the internal gear angle ${\uptheta } = {\upomega }_{2} {\text{t}}$ , where ${\upomega }_{2}$ is the internal gear speed, and the pressure change in the transition zone within ${\text{dt}}$ time is $dp_{2}$ . From the continuity equation of compressible fluids, then^27^

6 $$\sum q_{in} - \sum q_{out} = \frac{{dV_{2} }}{dt} + \frac{{V_{2} }}{{K_{e} }}\frac{{dp_{2} }}{{{\text{dt}}}}$$

In the equation: $K_{e}$ is the volume elastic modulus of hydraulic oil, $V_{2}$ is the volume of oil in the tooth cavity.

Substitute ${\text{q}}_{1}$, ${\text{q}}_{2}$, ${\text{q}}_{3}$, ${\text{q}}_{4}$ and ${\text{q}}_{5}$ into Eq. (6),

7 $${\text{q}}_{1} + {\text{q}}_{3} - {\text{q}}_{2} - {\text{q}}_{4} - {\text{q}}_{5} = \frac{{dV_{2} }}{dt} + \frac{{V_{2} }}{{K_{e} }}\frac{{dp_{2} }}{{{\text{dt}}}}$$

Due to the fixed volume of the tooth concave, $\frac{{dV_{2} }}{dt} = 0$. When the internal gear speed is ${\text{n}}_{2}$ , $\frac{{dp_{2} }}{{{\text{d}}t}} = 2\uppi n_{2} \frac{{dp_{2} }}{{{\text{d}}\upvarphi _{2} }}$ , then the non-linear differential equation of the pressure in the tooth concave area can be sorted out to obtain,

8 $$\frac{{dp_{2} }}{{{\text{d}}\uptheta }} = \frac{{K_{e} }}{{2\uppi {\text{n}}_{2} V_{2} }}\left\{ {\left[ {\frac{{bh_{{2i}}^{3} }}{{12\mu {\text{s}}_{{e2}} }} + \frac{{R_{{e2}} - R_{{i2}} }}{{6\mu {\text{s}}_{2} }}h_{{2f}}^{3} } \right](p_{g} - 2p_{2}) - \frac{{\theta h_{{2f}}^{3} }}{{6\mu ln\frac{{R_{i} }}{{R_{{z2}} }}}}p_{2} } \right\}$$

Set, $$A_{1} = \frac{{K_{e} }}{{2{\pi n}_{2} V_{2} }}$$, $$A_{2} = \frac{{bh_{2i}^{3} }}{{12\mu {\text{s}}_{e2} }}$$, $$A_{3} = \frac{{R_{e2} - R_{i2} }}{{6\mu {\text{s}}_{2} }}h_{2f}^{3}$$,$$A_{4} = \frac{{\theta h_{2f}^{3} }}{{6\mu ln\frac{{R_{i} }}{{R_{z2} }}}}$$.

Then, Eq. (8) can be simplified as

9 $$\frac{{dp_{2} }}{{{\text{d}}\uptheta }} = A_{1} \left[ {\left( {A_{2} + A_{3} } \right) (p_{g} - 2p_{2}) - A_{4} p_{2} } \right]$$

The Eq. (9) is the differential equation for the pressure change in the tooth concave area, and its initial conditions are:$\uptheta = 0$, $p_{2} = p_{g}$.

### Pressure distribution in the transition zone

**Figure 3:**
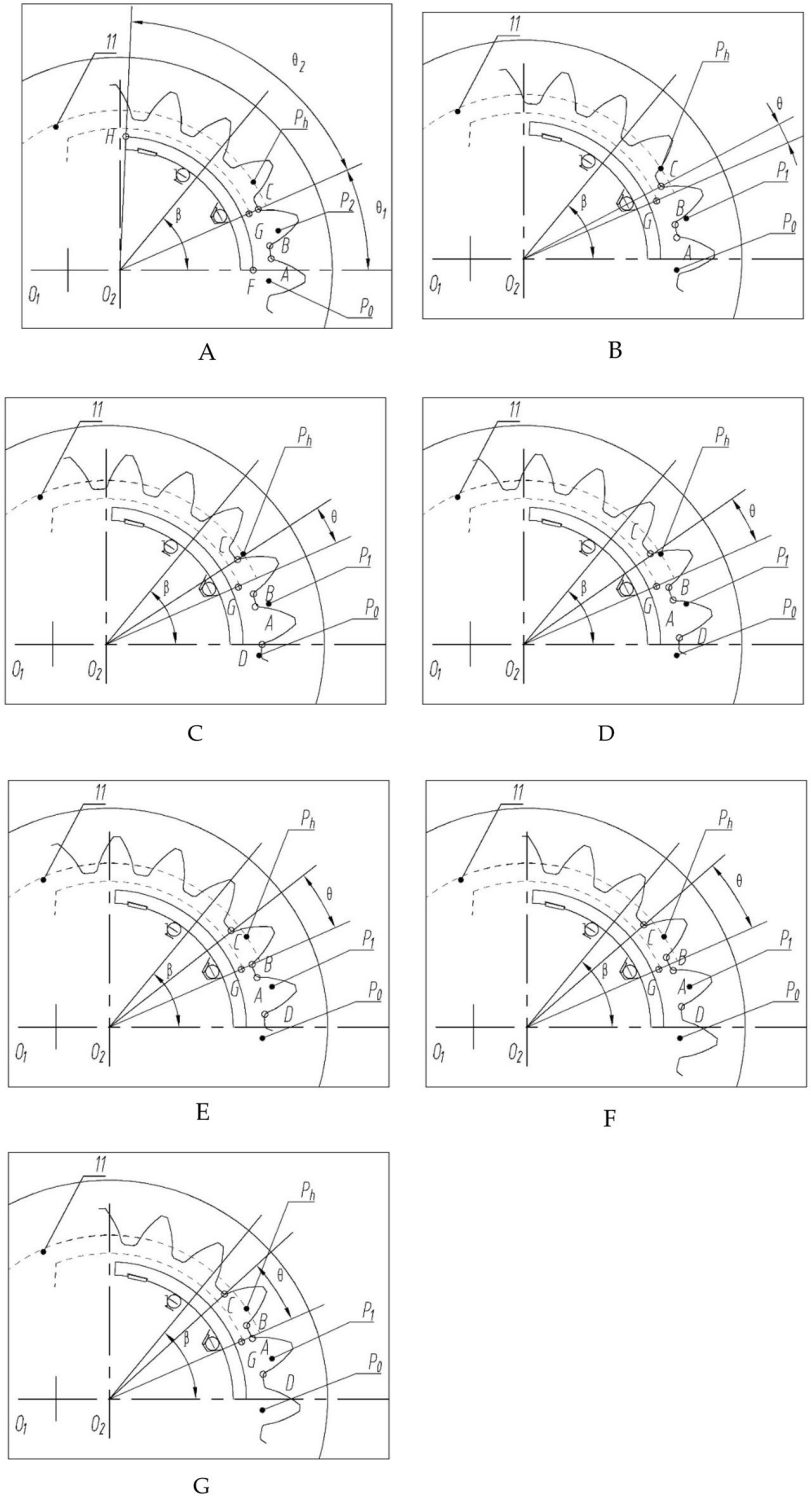
Pressure distribution in the transition zone: (**A**) Starting position, ${\uptheta } = 0$°. (**B**) Gear rotation angle ${\uptheta }\left( {0 < {\uptheta } < \theta^{\prime} - {{\uptheta^{\prime\prime}}}} \right)$; (**C**) Gear rotation angle ${\uptheta } = \theta^{\prime} - {{\uptheta^{\prime\prime}}}$; (**D**) Gear rotation angle ${{\uptheta }}\left( {\theta ^{\prime} - {{\uptheta ^{\prime\prime}}} < {{\uptheta }} < {{\uptheta ^{\prime}}}} \right)$; (**E**) Gear rotation angle ${\uptheta } = {{\uptheta^{\prime}}}$; (**F**) Gear rotation angle ${\uptheta }\left( {\theta^{\prime} < {\uptheta } < \theta^{\prime} + {{\uptheta^{\prime\prime}}}} \right)$; (**G**) Gear rotation angle ${\uptheta } = \theta^{\prime} + {{\uptheta^{\prime\prime}}}$.

As shown in Fig. 2, the included angle of a tooth concave on the tooth top circle is $\theta^{\prime}$, and the included angle of a gear tooth on the tooth top circle is ${\uptheta ^{\prime\prime}}$. The included angle of the outer lunar pressure block is $\theta_{1} + \theta_{2}$, the included angle of the tooth concave area is $\theta_{1}$, and the included angle of the high-pressure area is $\theta_{2}$. The sum of $\theta^{\prime}$ and ${\uptheta ^{\prime\prime}}$ is $\alpha$, $\alpha = \frac{2\pi }{z}$, $z$ is the number of teeth. The change period of pressure $p\left( \theta \right)$ in the transition zone is $\alpha$. The change in pressure $p\left( \theta \right)$ is shown in Fig. 3.

The initial position is the line connecting the endpoint of oil groove 11 and the center of circle O_2_, where point C of the tooth groove just passes through. The process of point C rotating angle ${\uptheta }\left( {0 < {\uptheta } < \theta^{\prime} - {{\uptheta^{\prime\prime}}}} \right)$.At this point, the tooth concave in the figure is in communication with the high-pressure chamber, and the distribution of $p\left( \theta \right)$ is,

10 $$p\left( \theta \right) = \left\{ \begin{gathered} p_{g} ,\uptheta \in \left( {\theta_{1} - \left( {\theta^{\prime} - \theta } \right),\theta_{1} + \theta_{2} } \right) \hfill \\ \frac{{\beta - \theta_{1} - \left( {\theta^{\prime} - \theta } \right) - {{\uptheta^{\prime\prime}}}}}{{{{\uptheta^{\prime\prime}}}}}\left( {p_{g} - p_{2} } \right),\uptheta \in \left( {\theta_{1} - \left( {\theta^{\prime} - \theta } \right) - {{\uptheta^{\prime\prime}}},\theta_{1} - \left( {\theta^{\prime} - \theta } \right)} \right) \hfill \\ 0,\uptheta \in \left( {0,\theta_{1} - \left( {\theta^{\prime} - \theta } \right) - \theta^{\prime \prime} } \right) \hfill \\ \end{gathered} \right.$$

${\upbeta }$ is the angle between the line connecting any point in the region and point O_2_ and the x-axis. The process of tooth concave point C rotating angle ${\uptheta }\left( {\theta^{\prime} - {{\uptheta^{\prime\prime}}} < {\uptheta } < {{\uptheta ^{\prime}}}} \right)$, and the distribution of $p\left( \theta \right)$ is,

11 $$p\left( \theta \right) = \left\{ \begin{gathered} p_{g} ,\theta \in \left( {\theta_{1} - \left( {\theta^{\prime} - \theta } \right),\theta_{1} + \theta_{2} } \right) \hfill \\ \frac{{\beta - \theta_{1} - \left( {\theta^{\prime} - \theta } \right) - \theta^{\prime\prime}}}{{\theta^{\prime\prime}}}\left( {p_{g} - p_{2} } \right),\theta \in \left( {\theta_{1} - \left( {\theta^{\prime} - \theta } \right) - \theta^{\prime\prime},\theta_{1} - \left( {\theta^{\prime} - \theta } \right)} \right) \hfill \\ p_{2} ,\theta \in \left( {\theta_{1} - \left( {2\theta^{\prime} - \theta } \right) - \theta^{\prime\prime},\theta_{1} - \left( {\theta^{\prime} - \theta } \right) - \theta^{\prime\prime}} \right) \hfill \\ \frac{\beta }{{\theta_{1} - \left( {2\theta^{\prime} - \theta } \right) - \theta^{\prime\prime}}}\left( {p_{2} - p_{0} } \right),\theta \in \left( {0,\theta_{1} - \left( {2\theta^{\prime} - \theta } \right) - \theta^{\prime\prime}} \right) \hfill \\ \end{gathered} \right.$$

The process of tooth concave point C rotating angle ${{\uptheta }}\left( {\theta ^{\prime} - {{\uptheta ^{\prime\prime}}} < {{\uptheta }} < {{\uptheta '}}} \right)$, and the distribution of $p\left( \theta \right)$ is,

12 $$p\left(\theta \right)=\left\{\begin{array}{l}{p}_{g},\theta \in ({\theta }_{1},{\theta }_{1}+{\theta }_{2})\\ \frac{\beta -{\theta }_{1}-\left({\theta }^{\prime}-\theta \right)-{\theta }^{{\prime\prime} }}{\left({\theta }^{\prime}-\theta \right)+{\theta }^{^{\prime\prime} }}\left({p}_{g}-{p}_{2}\right)\begin{array}{c}\\ ,\theta \in \left({\theta }_{1}-\left({\theta }^{\prime}-\theta \right)-{\theta }^{{\prime\prime} },{\theta }_{1}\right)\\ \end{array}\\ {p}_{2}\begin{array}{c}\\ ,\theta \in \left({\theta }_{1}-\left({2\theta }^{\prime}-\theta \right)-{\theta }^{{\prime\prime} },{\theta }_{1}-\left({\theta }^{\prime}-\theta \right)-{\theta }^{{\prime\prime} }\right)\\ \end{array}\\ \frac{\beta -{\theta }_{1}-\left({2\theta }^{\prime}-\theta \right)-2{\theta }^{^{\prime\prime} }}{{\theta }^{{\prime\prime} }}\left({p}_{2}-{p}_{0}\right),\theta \in [{\theta }_{1}-({2\theta }^{\prime}-\theta )-2{\theta }^{{\prime\prime} },{\theta }_{1}-({2\theta }^{\prime}-\theta )-{\theta }^{^{\prime\prime} }]\\ {p}_{0},\theta \in \left(0,{\theta }_{1}-\left({2\theta }^{\prime}-\theta \right)-2{\theta }^{{\prime\prime} }\right)\end{array}.\right.$$

## Design of isolation device

### Force on the external crescent block

**Figure 4:**
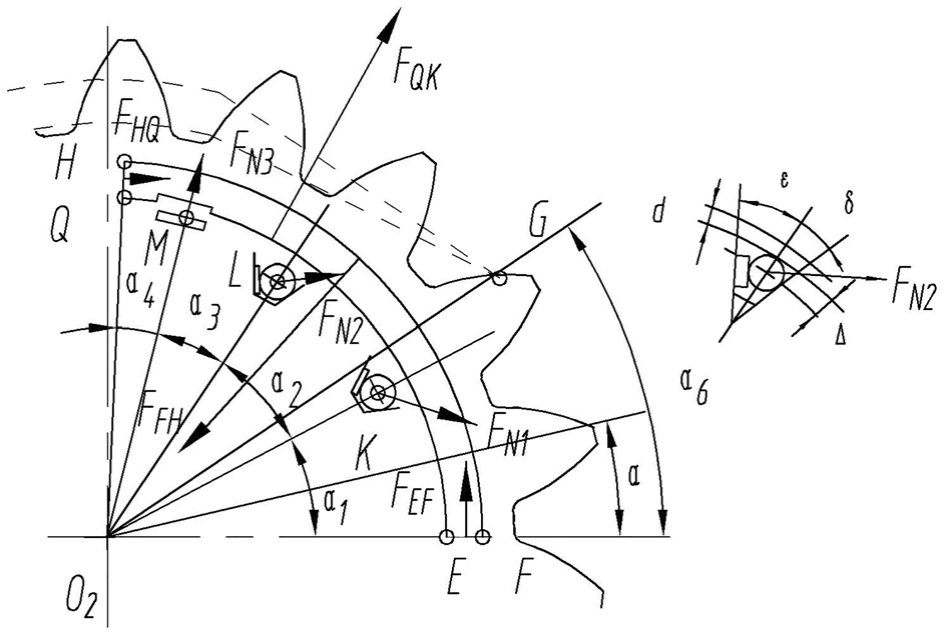
Hydrodynamic model of the outer crescent block.

The force analysis of the outer crescent block as the research object is shown in Fig. 4. The FH section is subjected to pressure in the transition zone, and the direction of the force always points towards the center of the circle ${{\text{o}}}_{2}$; the HQ section is subjected to high-pressure hydraulic pressure, and its direction is always perpendicular to the HQ section; high pressure oil is introduced into the oil chamber of Zone I of the QL section, pushing the crescent block radially outward, and the direction of force always points towards the center of the circle ${{\text{o}}}_{2}$; the EF section is subjected to a reaction force from the stop pin, and the direction of the force is always perpendicular to the pressure boundary line; Zones II and III are low-pressure zones, with point K subjected to the elastic force ${{\text{F}}}_{N1}$ of the rectangular compression spring 4 at the center of the circle; Point L and point M are subjected to the elastic force perpendicular to the baffle spring;the top circle radius of the inner gear is ${{\text{r}}}_{o2}$, the top circle radius of the outer gear is ${{\text{r}}}_{o1}$, the gear thickness is ${\text{b}}$, and the radial thickness of the outer crescent block is d, the theoretical clearance between the inner and outer crescent blocks is $\Delta$, the position angle of the baffle spring are $\upvarepsilon$, $\updelta$; the angle between the pressure boundary line and the X-axis is ${\alpha }_{0}$, the center angle between the second sealing rod and the pressure boundary line is ${\alpha }_{1}$, the center angle between the first sealing rod and the second sealing rod is ${\alpha }_{2}$, the center angle between the first baffle spring and the first sealing rod is ${\alpha }_{3}$.

Segmental analysis:

FH segment:

The change in pressure in the transition zone within a cycle.

13 $$\left\{ {\begin{array}{*{20}c} {F_{FHx} = r_{o2} b\mathop \int\nolimits_{0}^{{\theta_{1} + \theta_{2} }} p\left( \theta \right)cos\alpha d\alpha } \\ {F_{FHy} = r_{o2} b\mathop \int \nolimits_{0}^{{\theta_{1} + \theta_{2} }} p\left( \theta \right)sin\alpha d\alpha } \\ \end{array} } \right.$$

$${\text{H}}^{\prime } {\text{J}}$$$${\text{HQ}}$$ segment:

14 $$\left\{ {\begin{array}{*{20}c} {F_{HQx} = p_{h} r_{o2} \left( {\alpha_{3} + \alpha_{4} } \right)bcos\left( {\alpha_{0} + \alpha_{1} + \alpha_{3} + \alpha_{4} } \right)} \\ {F_{HQy} = p_{h} r_{o2} \left( {\alpha_{3} + \alpha_{4} } \right)bsin\left( {\alpha_{0} + \alpha_{1} + \alpha_{3} + \alpha_{4} } \right)} \\ \end{array} } \right.$$

$${\text{EF}}$$ segment:

15 $$\left\{ {\begin{array}{*{20}c} {F_{EFx} = F_{EF} cos\alpha_{0} } \\ {F_{EFy} = F_{EF} sin\alpha_{0} } \\ \end{array} } \right.$$

The elastic force of the rectangular compression spring is $F_{N1}$:

16 $$\left\{ {\begin{array}{*{20}c} {F_{N1x} = F_{N1} cos\left( {\alpha_{0} + \alpha_{1} + \alpha_{3} } \right)} \\ {F_{N1y} = F_{N1} sin\left( {\alpha_{0} + \alpha_{1} + \alpha_{3} } \right)} \\ \end{array} } \right.$$

The spring force of the first baffle is $F_{N2}$:

17 $$\left\{ {\begin{array}{*{20}c} {F_{N2x} = F_{N2} cos\left( {\alpha_{0} + \alpha_{1} + \alpha_{3} + \varepsilon } \right)} \\ {F_{N2y} = F_{N2} sin\left( {\alpha_{0} + \alpha_{1} + \alpha_{3} + \varepsilon } \right)} \\ \end{array} } \right.$$

The spring of the second baffle is $F_{N3}$:

18 $$\left\{ {\begin{array}{*{20}c} {F_{N3x} = F_{N3} cos\left( {\alpha_{0} + \alpha_{1} + \varepsilon } \right)} \\ {F_{N3y} = F_{N3} sin\left( {\alpha_{0} + \alpha_{1} + \varepsilon } \right)} \\ \end{array} } \right.$$

So, the combined external thrust force on the outer crescent block can be expressed as the following equation,

19 $$\left\{ {\begin{array}{*{20}c} {F_{Wx} = F_{FHx} + F_{HQx} + F_{EFx} + F_{N1x} + F_{N2x} + F_{N3x} } \\ {F_{Wy} = F_{FHy} + F_{HQy} + F_{EFy} + F_{N1y} + F_{N2y} + F_{N3y} } \\ \end{array} } \right.$$

### Force on the inner crescent block

**Figure 5:**
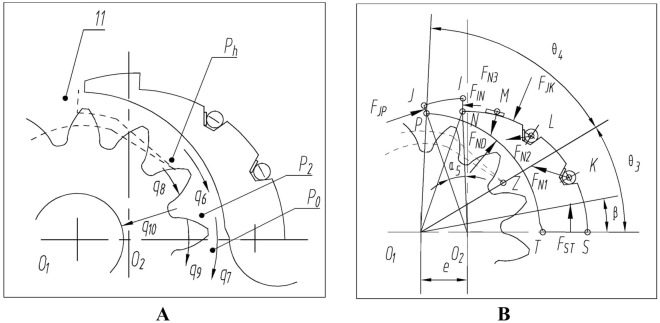
Mechanical model of the inner crescent block: (**A**) Geometric and hydrodynamic models of the tooth recess; (**B**) A hydrodynamic model of the inner crescent block.

The force analysis of the inner crescent block as the research object is shown in Fig. 5. The angle range of the internal crescent block is ${\theta }_{3}+{\theta }_{4}$, the included angle of the tooth concave area is ${\theta }_{3}$, and the included angle of the high-pressure area is ${\theta }_{4}$.The PT section is subjected to pressure in the transition zone, and the direction of the force always points towards the center of the circle ${{\text{o}}}_{1}$; The ST segment is subjected to the support reaction force ${F}_{ST}$ of the stop pin, and the direction of the force is always perpendicular to the pressure boundary line; The JP section is subjected to high-pressure hydraulic pressure ${F}_{JP}$ , with a direction perpendicular to the JP section; The IJ section is subjected to high-pressure hydraulic pressure ${F}_{IJ}$ and points towards the center of the circle ${{\text{o}}}_{2}$; The NI section is subjected to hydraulic pressure ${F}_{NI}$ and its direction is perpendicular to the NI section; The sizes of ${F}_{N1}$, ${F}_{N2}$ and ${F}_{N3}$ are the same as those of the external crescent, but the direction of force is opposite.

$${\text{PT}}$$ segment:

The flow into and out of the tooth concave area are respectively $q_{6}$,$q_{7}$, $q_{8}$,$q_{9}$ and $q_{10}$. Calculate the pressure in the concave area of the tooth according to the calculation method of the outer crescent, and similarly calculate the force $F_{PTx}$,$F_{PTy}$.

$${\text{ST}}$$ segment:

$F_{ST}$ is the reaction force of the pin support.

$${\text{JP}}$$ segment:

Applying the sine theorem in $\Delta O_{1} O_{2} P$

20 $$\frac{{O_{2} P}}{{sin\beta_{1} }} = \frac{e}{{sinO_{1} PO_{2} }} = \frac{{r_{o1} }}{{{\text{sin}}\left( {1.5\pi - \alpha_{1} - \alpha_{3} - \alpha_{3} - \alpha_{4} - \alpha_{5} } \right)}}$$

So, $${\text{JP}} = r_{o2} - O_{2} P$$, therefore

21 $$\left\{ {\begin{array}{*{20}c} {F_{{{\text{JP}}x}} = p_{h} \times JP \times dcos\left( {\alpha_{0} + \alpha_{1} + \alpha_{3} + \alpha_{4} + \alpha_{5} } \right)} \\ {F_{{{\text{JP}}y}} = p_{h} \times JP \times dsin\left( {\alpha_{0} + \alpha_{1} + \alpha_{3} + \alpha_{4} + \alpha_{5} } \right)} \\ \end{array} } \right.$$

$${\text{IJ}}$$ segment:

22 $$\left\{ {\begin{array}{*{20}c} {F_{{{\text{IJ}}x}} = r_{o2} b\mathop \int \nolimits_{{\alpha_{0} + \alpha_{1} + \alpha_{3} + \alpha_{4} }}^{{\alpha_{0} + \alpha_{1} + \alpha_{3} + \alpha_{4} + \alpha_{5} }} p_{h} \alpha_{5} cos\alpha d\alpha .} \\ {F_{{{\text{IJ}}y}} = r_{o2} b\mathop \int \nolimits_{{\alpha_{0} + \alpha_{1} + \alpha_{3} + \alpha_{4} }}^{{\alpha_{0} + \alpha_{1} + \alpha_{3} + \alpha_{4} + \alpha_{5} }} p_{h} \alpha_{5} sin\alpha d\alpha .} \\ \end{array} } \right.$$

$${\text{NI}}$$ segment:

23 $$\left\{ {\begin{array}{*{20}c} {F_{{{\text{NI}}x}} = p_{h} bd\left( {r_{o2} - d - \Delta } \right)cos\left( {\alpha_{0} + \alpha_{1} + \alpha_{3} + \alpha_{4} } \right)} \\ {F_{{{\text{NI}}y}} = p_{h} bd\left( {r_{o2} - d - \Delta } \right)sin\left( {\alpha_{0} + \alpha_{1} + \alpha_{3} + \alpha_{4} } \right)} \\ \end{array} } \right.$$

So, the combined external thrust force on the inner crescent block can be expressed as follows:

24 $$\left\{ {\begin{array}{*{20}c} {F_{Nx} = F_{PTx} + F_{STx} + F_{JPx} + F_{IJx} + F_{NIx} + F_{N2x} + F_{N3x} } \\ {F_{Ny} = F_{PTy} + F_{STy} + F_{JPy} + F_{IJy} + F_{NIy} + F_{N2y} + F_{N3y} } \\ \end{array} } \right.$$

### Calculation and design

When the gear pump is working, in order to reduce radial leakage, it is necessary to ensure that the inner and outer crescent blocks always fit the tooth top circle, and to prevent excessive jamming between the crescent blocks and the gear, the outer surface of the crescent and the gear must have lubrication properties^28–30^. Therefore, the pressure of the compensation chamber cannot be designed too small or too large. Therefore, it is necessary to reasonably design the angle $\mathrm{\alpha^{\prime}}({\mathrm{\alpha ^{\prime}}}={\alpha }_{3}+{\alpha }_{4})$ of the QL section of the compensation chamber formed by the inner and outer crescent blocks in the compensation device.

The compensation forces in the QL are equal in magnitude and opposite in direction, and in the design of high-pressure gear pumps, the forces ${F}_{N1}$,${F}_{N2}$ and ${F}_{N3}$ are much smaller than the hydraulic pressure in the transition zone^31–33^. Therefore, when establishing constraint programming, the sizes of ${F}_{N1}$,${F}_{N2}$ and ${F}_{N3}$ can be ignored to obtain ${F}_{EF}$ and ${F}_{ST}$.

During normal operation of the gear pump, the inner and outer EF and ST sections always adhere to the stop pins, ensuring that ${F}_{EF}\ge 0$ and ${F}_{ST}\ge 0$ are met.

Substitute its value into Eqs. (19) and (24) to obtain the X and Y components when the inner and outer lunar blocks rotate.

Calculate the average value $\overline{{F}_{WX}}$, $\overline{{F}_{WY}}$,$\overline{{F}_{NX}}$ and $\overline{{F}_{NY}}$ for each cycle.

Calculate the external thrust $F_{W} = \sqrt {\left( {\overline{{F_{WX} }} } \right)^{2} + \left( {\overline{{F_{WY} }} } \right)^{2} }$ and $F_{N} = \sqrt {\left( {\overline{{F_{{NX}} }} } \right)^{2} + \left( {\overline{{F_{{NY}} }} } \right)^{2} }$ of the inner and outer crescent blocks.

The established objective function is:

25 $$\left\{ {\begin{array}{*{20}l} {min\left[ {\left| {F_{W} - P_{h} \left( {r_{o2} - d} \right)b\alpha {^{\prime}}} \right| + \left| {F_{N} - P_{h} \left( {r_{o2} - d} \right)b\alpha {^{\prime}}} \right|} \right]} \\ {s.t.F_{EF} \ge 0,F_{ST} \ge 0} \\ {0 \le \alpha^{\prime} \le \alpha_{1} + \alpha_{2} + \alpha_{3} + \alpha_{4} } \\ \end{array} } \right.$$

## Experimental research

### Material and methods

The material of the inner and outer Crescent blocks is beryllium bronze, and its processing technology conditions refer to FED-STD-00153, Copper Base Alloy Casting, Chimical Combustion And Mechanial Properties issued on June 30, 1967.The material of the sealing rod is tetrafluoroethylene filled with 25% graphite, and its technical requirements and test methods refer to ISO23529, Rubber-General Process For Preparing And Condioning Test Pieces For Physical Test Methods, issued on October 15, 2010.The gear material is 20GrMnTi, and its mechanical properties meet the requirements of ISO4990, Steel Castings-General Technical Requirements, issued on November 1, 2003. The test method referred to the Rotodynamic Pumps Hydraulic Performance Acceptance Tests, ANSI/HI 14.6-2011, issued in 2011. Then, the testing process also referred to ISO9906, Rotodynamic Pumps-Hydraulic Performance Access Test -Grades 1 And 2, issued on May 1, 2012. The test bench is shown in Fig. 7E.

### Experimental results

**Figure 6:**
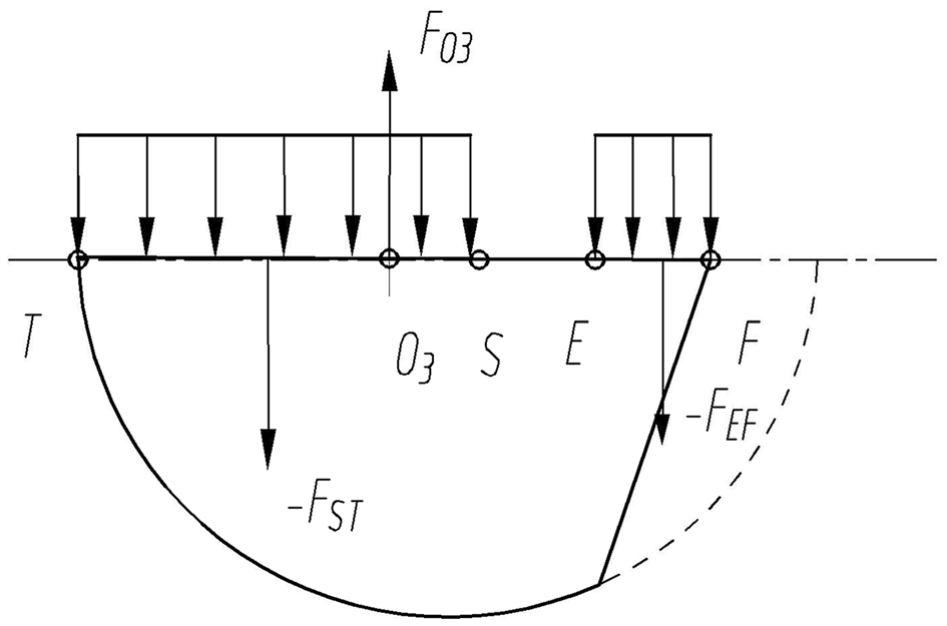
Support reaction model of the steering pin.

**Figure 7:**
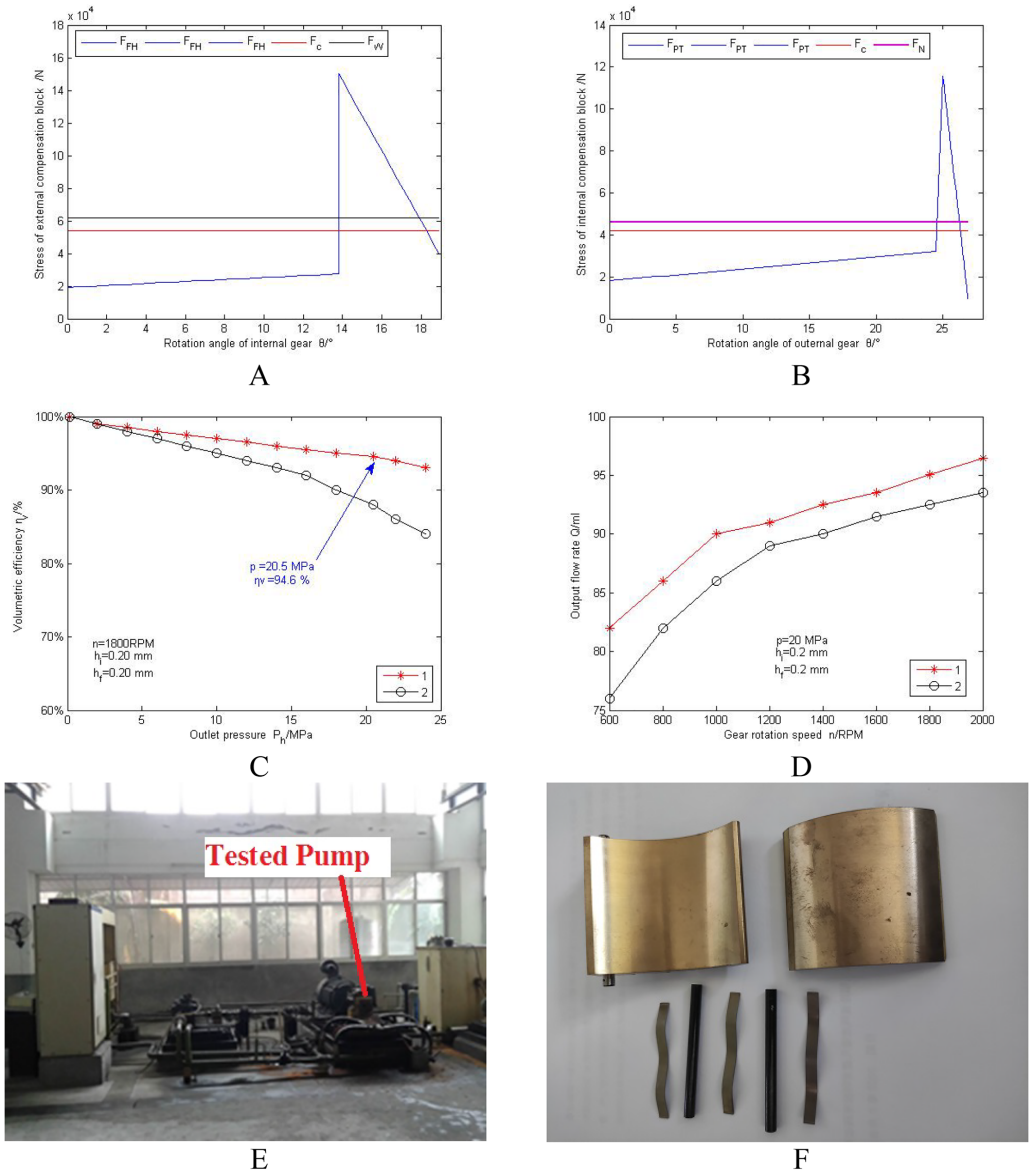
Experimental study of an internal gear pump: (**A**) The force variation curve of the external crescent block. (**B**) The force variation curve of the internal crescent block; (**C**) Volume efficiency curve with pressure; (**D**) Curve of flow changing with speed; (**E**) Bench testing; (**F**) Disassembled part diagram.

Taking the IPFY series internal meshing gear pump developed by the Hydraulic Parts Factory of Fuzhou University as the object (as shown in Fig. 6), with a theoretical displacement of 100.5 ml/r and a rated pressure of 20 MPa, experimental research was conducted. The design parameters : gear modulus ${\text{m}} = 3$, external tooth number $z_{1} = 13$, internal tooth number $z_{2} = 9$, pressure angle of 20°, tooth width thickness ${\text{b}} = 41$ mm, external gear modification coefficient $\xi_{1} = 0.432$, internal gear gear modification coefficient $\xi_{2} = 0.553$, gear pair center distance ${\text{e}} = 9.253$ mm, tooth top height coefficient $h_{a}^{*} = 1$ mm, top clearance coefficient $c^{*} = 0.25$ mm, external crescent tooth thickness ${\text{d}} = 2.6$ mm, initial clearance of internal and external crescent blocks $\Delta = 0.3$ mm, initial angle of pressure boundary 0°, top circle radius of internal teeth $r_{o1} = 23.8$ mm, the radius of the top circle of the outer gear tooth is $r_{o2} = 27.15$ mm, the center angle of the outer gear tooth groove on the top circle is $\theta_{1}^{\prime} = 25^\circ$, the center angle of the outer gear tooth groove on the top circle is $\theta^{\prime\prime}_{1} = 2.7^\circ$, the center angle of the inner gear tooth groove on the top circle is $\theta_{2}^{\prime} = 13.8^\circ$, the center angle of the inner gear tooth groove on the top circle is $\theta^{\prime\prime}_{2} = 5.1^\circ$, the center angle of the outer crescent boundary arc length is $\alpha_{1} + \alpha_{2} + \alpha_{3} + \alpha_{4} = 75.6^\circ$, and the center angle of the inner crescent boundary arc length is $\theta_{3} + \theta_{4} = 83.1^\circ$, The installation angle of the first and second baffle springs is ${\upvarepsilon } = 30.9$, ${\updelta } = 18.4$, taking $F_{N1} = F_{N2} = F_{N3} = 250 N$ and $\alpha_{1} = \alpha_{2}$,$\alpha_{3} = \alpha_{4}$ respectively. Use MATLAB programming to calculate the magnitude of the external and internal thrust $F_{W}$ and $F_{N}$ of the inner and outer crescent blocks when rotating through a tooth slot and a gear tooth. Constrained optimization of the objective function was performed using the optimization tool fminbnd, and $\alpha^{\prime} = 31.23^\circ$ was obtained through analysis. $F_{c}$ is the compensating force. The Fig. 7A,B shows that during the process of gear rotation, the force on the crescent block shows a sudden and abrupt change. The variation of $F_{FH}$ with rotation angle is consistent with reference^34^. The variation of $F_{PT}$ with rotation angle is consistent with reference^35^. The size of the compensation force $F_{{\text{C}}}$ is similar to the force $F_{w}$ and $F_{N}$ on the crescent block, which is consistent with the conclusion drawn in reference^36^.

Under the environmental conditions of using L-HM46 hydraulic oil, with an inlet pressure of 0.13 MPa and an outlet pressure of 20.5 MPa, a test oil temperature of $50\pm 4\mathrm{^\circ{\rm C} }$, and a rated speed of 1800 RPM. The volumetric efficiency reached 94.6%, and there were no abnormal wear marks on the surface of the inner and outer crescent blocks. The parts are shown in Fig. 7F.

### Discussion of results

The experiment set up a control group. The gear pump in control group 2 adopts a fixed crescent plate structure, with an axial and radial clearance of 0.2 mm. Pump 1 and pump 2 have the same theoretical displacement. Figure 7C shows that gear pumps with compensated crescent plates have higher volumetric efficiency under high pressure; Fig. 7D shows that as the speed changes, pump 1 has a higher flow output.

## Conclusion

Based on the actual working conditions of the tooth groove and gear teeth, this paper establishes a geometric model of the radial compensation device for the internal meshing gear pump. By adopting the technical measure of reducing the sealing area of the crescent block to a tooth concave area, a fluid dynamics model of the crescent block was established, and the pressure variation with gear rotation in the transition zone was derived. Then, establish a design model to obtain the optimization angle of the compensation mechanism. These works can provide reference for the design and optimization of high-pressure internal meshing gear pumps, and also contribute to a deeper understanding of the lubrication characteristics of compensation mechanisms.

1. Taking the radial compensation device of the high-pressure internal meshing gear pump as the research object, the force analysis of the inner and outer crescent blocks was carried out. A design model was established during the gear rotation cycle, and based on this, fminbnd constraint optimization was carried out to obtain the compensation chamber angle, as well as the angle positions of the baffle spring, sealing rod, and rectangular compression spring.
2. Draw the curve of the hydraulic external thrust of the inner and outer crescent blocks as a function of the rotation angle. When passing through a tooth groove, the external thrust increases linearly, while when passing through a tooth, the external thrust decreases linearly.
3. Experimental research shows that the internal meshing gear has an output efficiency of 94.6% at an output pressure of 20.5 MPa, a speed of 1800 RPM, and a compensation chamber angle of ${\alpha }^{\prime}=31.23^\circ$. It operates normally without any phenomenon of jamming or locking. The feasibility and correctness of the model have been verified through practical examples.

## Deficiencies and future prospects

Due to the wide scope of modeling work, parameter variables such as material forming, machining errors, assembly tolerances, and cold and hot deformation of each material were not considered. The energy loss of the transmission medium in the circuit has not been considered. The research topic still has further research value. In the future, considering multiple factors, the accuracy of design models can continue to be improved. Verify the model by testing the outlet pressure of the pump and the leakage of the friction pair. Further increase the operating pressure of the internal meshing gear pump.

## Data Availability

The datasets used and/or analysed during the current study are available from the corresponding author on reasonable request.
